# Severity-dependent fronto-cingulate network compensation of immune-dependent caudate-insula dysconnectivity in bipolar II depression

**DOI:** 10.21203/rs.3.rs-8945790/v1

**Published:** 2026-03-24

**Authors:** Changjian Qiu, Yuan Cao, Huan Sun, Meng Li, Qiannan Zhao, Ling-Yu Huang, Xiaoqin Zhou, Huaiqiang Sun, Paulo Lizano, Martin Walter

**Affiliations:** Jena Uiversity Hospital; West China Hospital of Sichuan University; Jena University Hospital

**Keywords:** bipolar II depression, functional connectivity, inflammation, default mode network, caudate, insula

## Abstract

Depressive symptoms in bipolar II depression (BDII-D) arise from brain dysconnectivity within medial prefrontal cortex (mPFC)-centered networks, in interaction with peripheral inflammatory alterations. This study aimed to characterize structure-derived functional connectivity (FC) changes in BDII-D and to determine how dysconnectivity relates to depression severity and inflammatory markers, particularly interleukin (IL)-1β. A total of 147 individuals with BDII-D and 150 healthy controls underwent fMRI, peripheral inflammation, and clinical symptom assessments. Based on prior structural alterations in BDII-D, seed-based FC changes were examined. Partial correlation analyses with multiple comparisons correction evaluated associations among altered FC, inflammatory cytokines, psychiatric symptoms, and clinical profiles. A moderation analysis tested whether IL-1β moderated the relationship between altered FC and depressive symptoms in BDII-D. Greater depressive symptoms in BDII-D were associated with higher caudate-insula and right triangular inferior frontal gyrus-bilateral superior frontal gyrus (SFG) connectivity, as well as lower medial SFG-posterior cingulate cortex (PCC) connectivity. IL-1β moderated the relationship between caudate-insula connectivity and depressive symptoms. The association between lower medial SFG-PCC connectivity and greater depressive severity was absent in BDII-D with HAMD < 17 (mild depressive symptoms) but significant in those with HAMD ≥ 17(moderate-to-severe depressive symptoms). Lower left middle frontal gyrus-right lateral superior lateral occipital gyrus connectivity was significantly associated with higher psychotic positive symptoms. Depressive symptoms in BDII-D reflect inflammation-related dysconnectivity across emotion and sensory networks. mPFC alterations may indicate adaptive processes and a potential neuromodulation target, while frontal-visual dysconnectivity is related to psychotic symptoms, suggesting a role of visual processing in the pathophysiology of BDII-D.

## Introduction

Bipolar type II disorder with depressive episodes (BDII-D) is a highly prevalent and clinically challenging condition, in which depressive episodes predominate over hypomania, leading to frequent relapses and a high risk of misdiagnosis as unipolar depression [1]. Evidence from neuroscience and lesion-based network mapping studies have consistently implicated the medial prefrontal cortex (mPFC), dorsolateral prefrontal cortex (DLPFC), striatum, and insula as key regions of the neural circuitry underlying depressive symptomatology [2–5]. Notably, these regions do not function in isolation; rather, focal lesions or dysfunctions in anatomically distinct regions often map on shared functional networks associated with depression, underscoring that brain alterations linked to depressive symptoms are more appropriately understood at the connectivity or network level than as isolated regional abnormalities [6]. Prior evidence, including our previous findings, indicated that depressive symptoms in BDII-D are shaped by the interaction between brain dysconnectivity and peripheral inflammation [7–9]. Therefore, based on the gray matter alterations identified in our previous study, it is necessary to further examine the corresponding functional connectivity (FC) changes and to clarify how these FC alterations relate to depressive symptom severity and peripheral inflammatory markers in BDII-D.

Although the DLPFC has been historically targeted in depression due to its role in cognitive function and its accessibility for non-invasive stimulation, the evidence from affective neuroscience indicated that the mPFC function as a central hub in the neural circuitry underlying depressive mood states [10, 11]. In addition to depression, our study revealed structural alterations not only within regions related to the DLPFC, but also in regions associated with the mPFC in BDII-D [12]. This coexistence of structural changes across both lateral and medial prefrontal regions raises an important question: whether FC alterations linked to symptom severity in BDII-D are preferentially anchored to DLPFC-related structural changes, or instead to those involving the mPFC? Moreover, lesion-based network studies have demonstrated that depressive symptoms cannot be sufficiently explained by focal prefrontal abnormalities alone. Instead, lesions distributed across anatomically distinct regions, extending from prefrontal cortices to other cortical and subcortical regions, including dorsal anterior cingulate, inferior parietal cortex, striatum, and insula, have been shown to map onto shared functional networks implicated in emotional regulation [13]. Specifically, DLPFC and posterior cingulate cortex (PCC) connectivity has been repeatedly implicated in melancholic features of depression [14, 15].While alterations involving mPFC-PCC connectivity have been more closely linked to ruminative symptom dimensions [16, 17]. Moreover, abnormalities in PFC-limbic circuits as well as the anterior cingulate cortex (ACC)-insular circuits have also been observed in depression, underscoring the role of affective processing networks [18, 19]. These findings indicated a network-level perspective in which different depressive symptom dimensions might be related to distinct brain networks. Consistent with these network-level insights, we observed brain structural alterations in regions beyond the prefrontal cortex, including the insula and caudate, which were related to depressive symptoms and emotional abuse in BDII-D [12]. Therefore, characterizing the FC patterns associated with these structural alterations and their relationships with symptoms in BDII-D may help to clarify the network-level substrates relevant to different neuromodulation strategies.

Increasing evidence suggested that depression is not only associated with alterations in large-scale brain networks but are also tightly coupled with immune and inflammatory processes that can influence brain structure and function [20]. Peripheral inflammatory markers, including C-reactive protein (CRP), interleukin-6 (IL-6), tumor necrosis factor-α (TNF-α), and interleukin-1β (IL-1β) were found to be associated with depressive symptom severity [21, 22]. These cytokines are thought to affect central nervous system function through disruption of blood-brain barrier integrity, activation of microglia, modulation of monoaminergic neurotransmission, and dysregulation of the hypothalamic-pituitary-adrenal axis, thereby contributing to alterations in neural circuits implicated in mood regulation [20]. Among these inflammatory mediators, IL-1β plays a particularly important role in both major depressive disorder (MDD) and BD, as it is produced not only by peripheral immune cells but also by glial cells and neurons, enabling it to directly influence synaptic plasticity, neurotransmitter metabolism, and stress-related neuroendocrine responses [23, 24]. Additionally, our functional activity-based FC study has demonstrated that IL-1β moderated the relationships between connectivity of the middle frontal gyrus with the medial ACC and insula and depressive symptom severity in BDII-D, further highlighting IL-1β as a central inflammatory mediator linking peripheral immune activation to brain network dysfunction in depressive states [7]. However, it remains unclear whether any peripheral inflammatory markers, particularly IL-1β, are consistently associated with alterations in structural-based connectivity and depressive symptoms in BDII-D, warranting further investigation.

Based on these considerations, the present study builds directly on our previous findings of gray matter reductions in BDII-D by using these structurally altered regions as regions of interest (ROIs) to examine whether corresponding abnormalities in FC can be identified. Rather than focusing on isolated regions, we used a network-level approach to investigate how structural alterations may give rise to dysfunctional connectivity within mood-related circuits. We hypothesized that regions showing gray matter reduction, especially the PFC with an emphasis on the mPFC, ACC, insula, and subcortical nuclei, would show FC alterations related to psychiatric symptom in BDII-D. In addition, given our previous FC findings [7], we further examine whether peripheral inflammatory markers, with a particular focus on IL-1β, would again moderate the relationships between these connectivity alterations and depressive symptoms in BDII-D.

## Methods

This cross-sectional study was approved by the Medical Research Ethics Committee of West China Hospital following the Declaration of Helsinki [25]. All participants signed a written informed consent form prior to participation.

A total of 150 BDII-D individuals and 155 HCs were recruited between May 2020 and August 2022. Among them, 3 BDII-D individuals and 5 HCs who didn’t complete resting state fMRI scans were excluded from final analyses. Diagnostic assessments were conducted independently by two psychiatrists using the Structured Clinical Interview for The Diagnostic and Statistical Manual of Mental Disorders, Fifth Edition (SCID-5). Individuals with BDII-D completed the 17-item Hamilton Depression Scale (HAMD) [26], Hamilton Anxiety Scale (HAMA) [27], Positive and Negative Syndrome Scale (PANSS) [28] to assess depressive, anxiety, and psychotic symptoms, respectively. Suicide ideation intensity was evaluated using the Columbia-Suicide Severity Rating Scale (C-SSRS) [29]. Additionally, individuals with BDII-D and HCs completed the Childhood Trauma Questionnaire (CTQ) to assess exposure to childhood trauma before the age of 16 [30, 31]. Sleep and circadian rhythm characteristics were evaluated using the Morningness-Eveningness Questionnaire (MEQ), Epworth Sleepiness Scale (ESS), Pittsburgh Sleep Quality Index (PSQI), and the Biological Rhythms Interview of Assessment in Neuropsychiatry (BRIAN).

Blood samples were collected from individuals with BDII-D and analyzed for inflammatory markers, including IL-6, IL-1β, TNF-α, and CRP. Routine hematological parameters, including absolute counts of lymphocytes, monocytes, neutrophils, platelets, and white blood cells (WBCs), were also obtained. The neutrophil-to-lymphocyte (NTL), monocyte-to-lymphocyte (MTL), and platelet-to-lymphocyte (PTL) ratios were calculated as indirect markers of systemic inflammation. The inclusion criteria of participants, the evaluation of patients’ psychotropic medication treatment, and the measures of inflammatory cytokines are described in the Supplementary Method 1.1–1.3 and our previous studies[12].

### MRI Acquisition and Preprocessing

Neuroimaging was performed on a 3.0 T magnetic resonance scanner (Siemens 3.0 T Trio Tim, Germany) with a 32-channel phased-array head coil at West China Hospital.

The 3D T1-weighted images were acquired using a 3D-magnetization prepared rapid acquisition gradient echo sequences (3D-MPRAGE) with the following parameters: repetition time/echo time (TR/TE) = 2400/2.01ms; inversion time = 1000 ms; flip angle = 8°; slice thickness = 0.8 mm without gap; matrix = 320×320; field of view (FOV) = 256 ×256 mm^2^; voxel size = 0.8 × 0.8 × 0.8 mm^3^.

The functional MRI (fMRI) was acquired using a multiband gradient-echo planar imaging. with the following parameters: TR/ TE = 700/37.8 ms; flip angle = 52°; slice thickness = 2.1 mm; slice gap = 2.1 mm, matrix size = 100 × 100; FOV = 210 × 210 mm^2^.

The image quality was checked by MRI Quality Control tool (MRIQC, https://github.com/poldracklab/mriqc). The fMRI preprocessing procedure followed the Human Connectome Project (HCP) minimal preprocessing pipeline [32], which has been described in our previous studies and is shown in Supplementary Method 1.4 [7, 12].

### Statistical analysis

To assess differences in demographic and clinical characteristics between individuals with BDII-D and HCs, a variety of statistical tests were employed. For sociodemographic data, independent t-tests were applied to normally distributed variables, Mann-Whitney U tests were used for variables that did not follow a normal distribution, and chi-square tests were used for categorical variables. Additionally, log transformations were applied to inflammatory cytokines to ensure conformity with a normal distribution. All statistical analyses were conducted using R software version 4.3.2 (https://www.rstudio.com).

### Voxel-based morphometry (VBM) seed-based FC

Seed regions for FC analysis were derived from our previous VBM study, in which gray matter regions showing significant differences between individuals with BDII-D and HCs were identified. These regions were saved as binary brain masks and used as ROIs. Seed-based FC analyses were performed using the CONN toolbox (version 22a), implemented in Statistical Parametric Mapping (SPM12; https://www.fil.ion.ucl.ac.uk/spm) within MATLAB 2022b [33].

At the first level, Pearson’s correlation coefficients were computed between the mean time series of each seed ROI and the time series of all other voxels across the brain, generating individual FC maps. These correlation coefficients were subsequently transformed into normally distributed Z-scores using Fisher’s r-to-z transformation for second-level analyses.

At the group level, voxel-wise general linear models (GLMs) were applied to identify brain regions showing significant FC differences between the BDII-D and HC groups. Age, sex, education level, and medication load were controlled as covariates. Statistical significance was determined using a voxel-level cluster-forming threshold of p < 0.001, combined with a cluster-level false discovery rate (FDR)-corrected threshold of p < 0.05 [34].

### Partial Correlation analysis

Partial Pearson and Spearman correlation analyses were conducted with age, sex, education level, and medication load included as covariates. The covariates were selected based on prior knowledge and differences between BDII-D and HCs [12]. The associations between significant brain FC values and peripheral inflammatory markers (IL-1β, IL-6, TNF-α, and CRP), indirectly inflammatory markers (WBC, NTL ratio, MTL ratio, and PTL ratio), psychiatric symptoms, and clinical profiles were examined in BDII-D group. When group differences in FC between BDII-D and HCs and their associations with depressive severity within the BDII-D group consistently indicated that greater deviation from HC levels was associated with lower HAMD scores, further subgroup analyses were conducted using a cut-off score of 17 on the HAMD to stratify BDII-D into mild and moderate-to-severe depression subgroups. The same partial correlations analyses were also performed to assess associations between significant brain FC values and childhood adversity, as well as sleep and circadian rhythm characteristics, in both BDII-D and HCs. To control for multiple comparisons, p-values were adjusted using the false discovery rate (FDR) correction according to the Benjamini-Hochberg procedure, and the corresponding q-values are reported [35]. The degrees of freedom and the number of independent tests were also calculated (Supplementary Method 1.5).

### Moderation analysis

Based on the partial correlation results described above and our previous finding that IL-1β moderates the relationship between brain functional alterations and depressive symptoms [7], moderation analyses were conducted to further examine whether IL-1β similarly moderated the associations between VBM-derived FC alterations and depressive symptom severity. Bootstrapping with 5,000 resamples was applied to estimate 95% confidence intervals (CIs) for the moderation effects. Model diagnostics were systematically evaluated, including outlier detection and assumptions of multicollinearity, autocorrelation, normality, and homoscedasticity. Specifically, outliers were assessed using the Bonferroni test; multicollinearity was examined using variance inflation factors (VIFs); autocorrelation was evaluated using the Durbin-Watson statistic; normality of residuals was tested using the Shapiro-Wilk test; and homoscedasticity was assessed using the Breusch-Pagan test. The moderation model included age, sex, education level, and medication load as covariates. The analyses were performed in R using the “interactions”, “car”, and “stats” package.

## Results

### Demographic and clinical data

The demographic and clinical profiles of 147 BDII-D and 150 HCs are shown in Table 1. BDII-D and HCs showed significant differences in age (Cohen’s d = −0.39, p < 0.001) and education levels (Cohen’s d = −1.15, p < 0.001).

Compared with HCs, individuals with BD II-D exhibited significantly higher scores on the ESS (Cohen’s d = 0.86, p < 0.001), PSQI (Cohen’s d = 2.59, p < 0.001), and BRIAN total (Cohen’s d = 2.23, p < 0.001) and subscale measures, however, no significant group difference was observed in chronotype preference as indexed by the MEQ (Cohen’s d = 0.1, p = 0.80). Additionally, BDII-D showed significantly higher CTQ total scores (Cohen’s d = 1.53, p < 0.001) and CTQ subdomains including emotional abuse (Cohen’s d = 1.25, p < 0.001), physical abuse(Cohen’s d = 0.90, p < 0.001), sexual abuse(Cohen’s d = 0.57, p < 0.001), emotional neglect(Cohen’s d = 1.38, p < 0.001), and physical neglect(Cohen’s d = 1.26, p < 0.001).

### VBM seed-based FC differences between BDII-D and HCs

The ROIs were defined based on a previous VBM study and included the right inferior cerebellum; right medial superior frontal gyrus (mSFG); right orbital middle frontal gyrus (orbital MFG); right inferior frontal gyrus, triangular part (triangular IFG); left opercular IFG; right insula; left middle temporal gyrus (MTG); left gyrus rectus; bilateral temporal poles; bilateral MFG; bilateral orbital IFG; and bilateral caudate nuclei. Among these ROIs, compared with HCs, individuals with BDII-D presented significant FC patterns in four regions: the left caudate, left MFG, right triangular IFG, and right mSFG.

**Figure 1:**
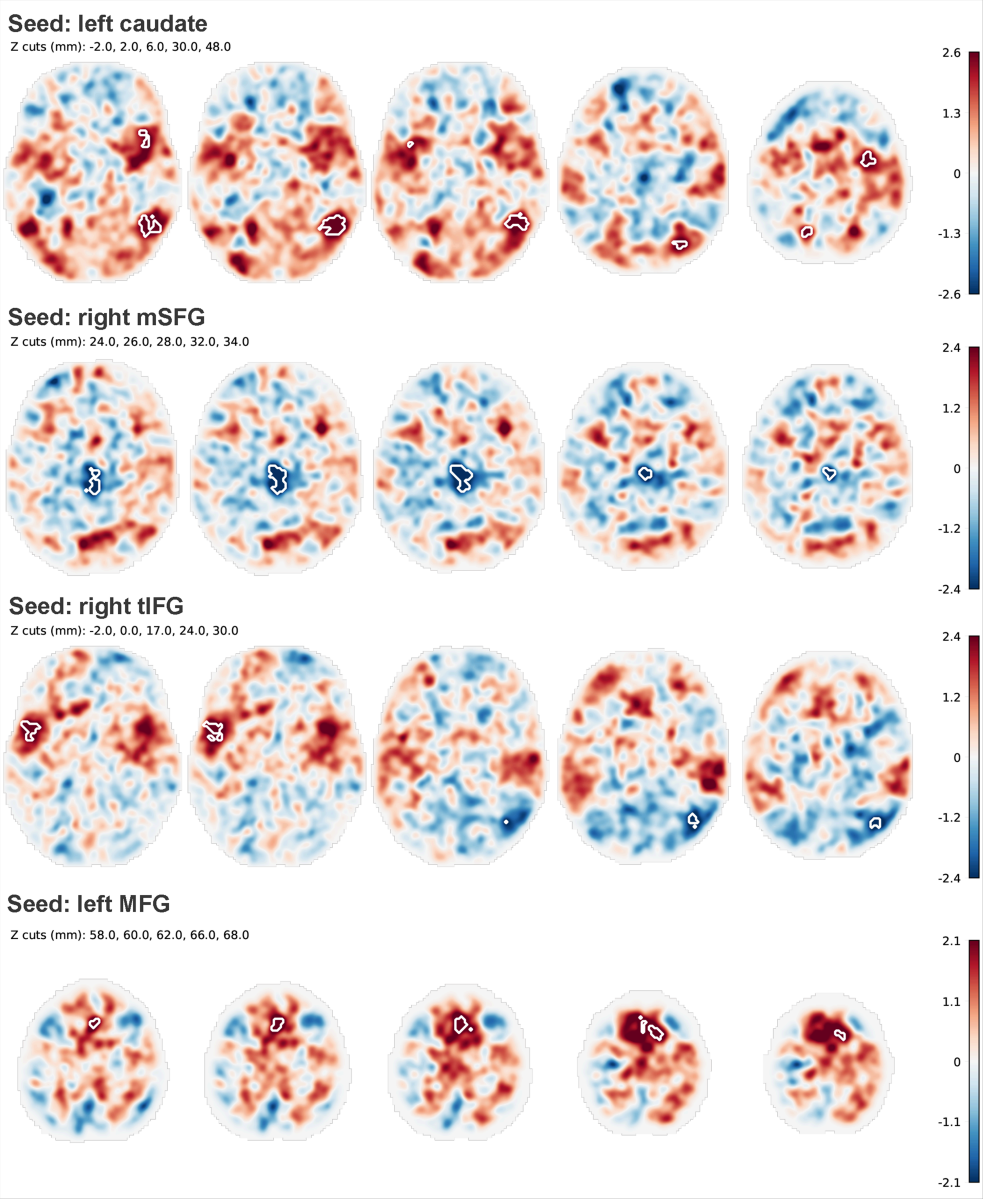
Seed based functional connectivity (FC) differences between individuals with BDII-D and HCs. The seeds with significant FC included: left caudate, left middle frontal gyrus (MFG), right triangular inferior frontal gyrus (triangular IFG), and right medial superior frontal gyrus (mSFG). **Left caudate:** significant higher FC were found between left caudate and right temporo-occipital gyrus, left lateral superior occipital gyrus (lateral SOG), right precentral gyrus, and left insula gyrus. **Left MFG:** significant higher FC were found between the left MFG and left insula gyrus, while lower FC were found with right lateral SOG. **Right triangular IFG:** significant higher FC were found between the right IFG triangular and bilateral SFG. **Right mSFG:** significant lower FC was found between right mSFG and posterior cingulate gyrus. Higher FC was shown in red, lower FC was shown in blue. The color bar indicated T values. **Abbreviations:**BDII-D-bipolar II depression; HCs-healthy controls.

Using the left caudate as the seed region, significantly higher FC was observed with the right temporal-occipital gyrus (MNI: 50, −58, 2; cluster size = 200; *p*_fdr_ < 0.001), left superior lateral occipital gyrus (lateral SOG) (MNI: −20, −66, 40; cluster size = 57; *p*_fdr_ = 0.017), right precentral gyrus (MNI: 34, −6, 48; cluster size = 45; *p*_fdr_ = 0.036), and left anterior insula (MNI: −32, 10, 10; cluster size = 40; *p*_fdr_ = 0.046). Using the right medial SFG as the seed, BDII-D individuals showed significantly lower FC with the posterior cingulate cortex (PCC) (MNI: 2, −20, 25; cluster size = 167; *p*_fdr_ < 0.001). When the right triangular IFG was used as the seed, significantly higher FC was detected with the bilateral SFG (MNI: 2, 14, 62 and − 2, 14, 62; *p*_fdr_ = 0.017). Using the left MFG as the seed region, significantly higher FC was identified with the left insula (MNI: −52, 8, 0; cluster size = 63; *p*_fdr_ = 0.01), whereas significantly lower FC was observed with the right lateral SOG (MNI: 40, −72, 26; cluster size = 58; *p*_fdr_ = 0.01) (Fig. 1 & Supplementary Table 1).

### Partial Correlations between altered FC and clinical features in BDII-D

**Figure 2:**
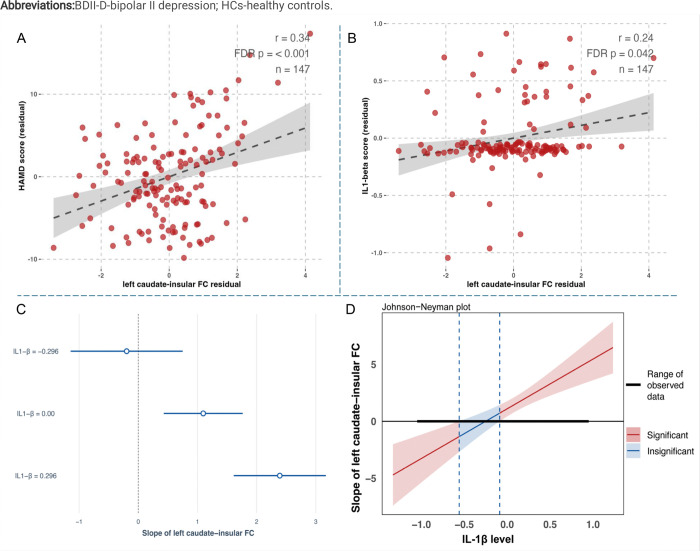
Partial correlation and moderation analysis among left caudate-insular functional connectivity (FC), depressive symptoms, and IL-1β in BDII-D group. (A) Higher caudate-insular FC value was significantly associated with higher HAMD score; (B) Higher caudate-insular FC value was significantly associated with higher IL-1β level; (C&D) Moderation analyses showed a moderating role of IL-1β in the associations between left caudate-insular FC and depressive symptoms. The age, sex, education level, and medication load were controlled in partial correlation and moderation analysis. FDR corrected q-values were presented in partial correlation analysis. **Abbreviations:**BDII-D-bipolar II depression; IL-interleukin.

In BDII-D population, higher FC value between left caudate and left insular was significantly associated with higher HAMD (r = 0.34, FDR q < 0.001) and higher IL-1β (r = 0.24, FDR q = 0.042) (Fig. 2). Additionally, higher FC values between right triangular IFG-bilateral SFG were significantly related to higher HAMD scores (right SFG: r = 0.28, q = 0.002; left SFG: r = 0.21, q = 0.023), while lower FC values between left MFG-right lateral SOG was significantly associated with higher PANSS positive scores (r = −0.29, q = 0.017) (Supplementary Fig. 1).

**Figure 3:**
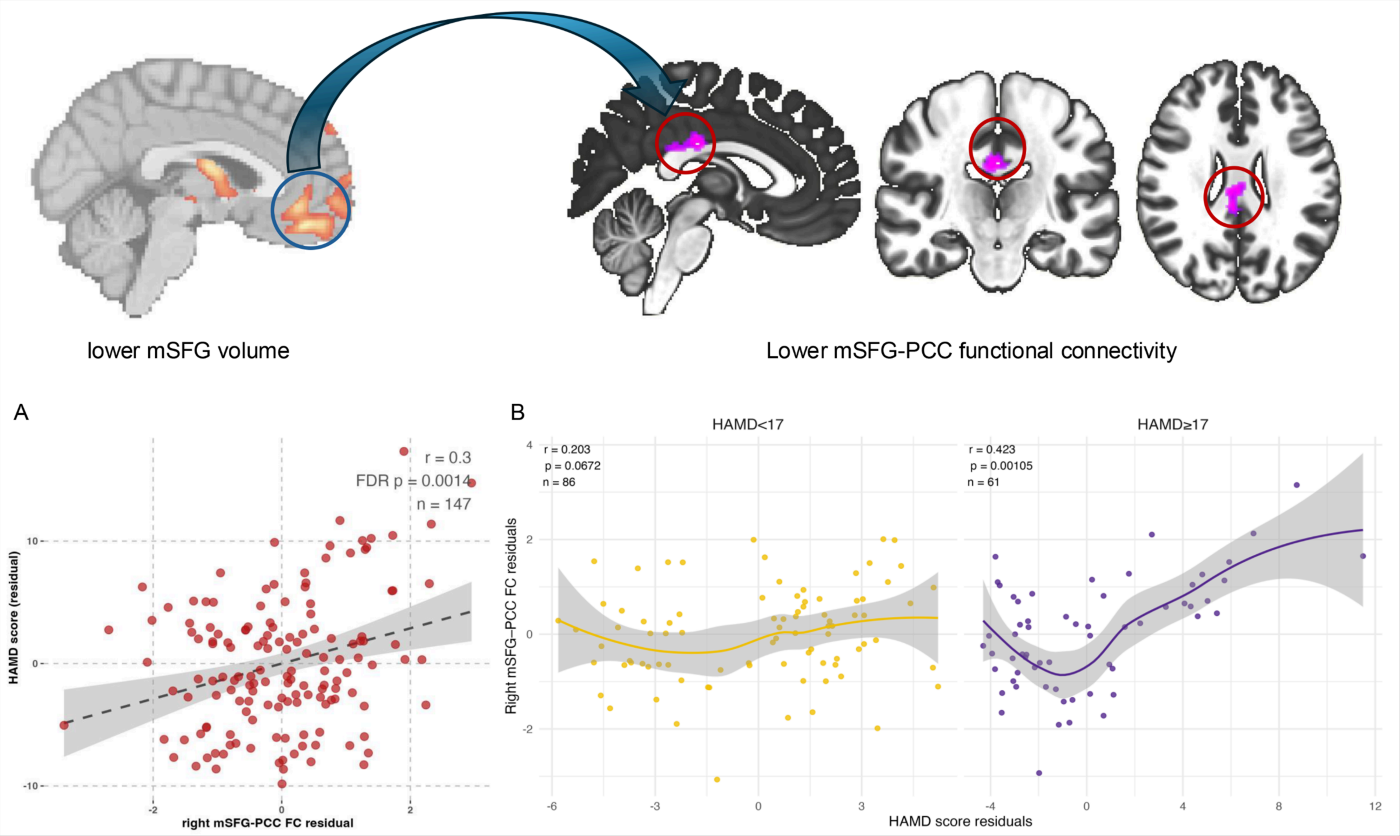
Partial correlation between right medial superior frontal gyrus (mSFG)-posterior cingulate cortex (PCC) functional connectivity (FC) and depressive symptoms in BDII-D. (A) higher right mSFG-PCC FC value was significantly associated with higher HAMD score in BDII-D; (B) Stratified individuals with BDII-D into mild and moderate-to-severe depression subgroups, the mSFG-PCC and HAMD association was nonsignificant in the mild subgroup but significant in the moderate-to-severe subgroup. **Abbreviations:**BDII-D-bipolar II depression; HAMD-17-item Hamilton Depression Scale.

However, our study showed a counterintuitive finding that lower FC value between right mSFG and PCC was significantly associated with lower HAMD (r = 0.30, q = 0.001) (Fig. 3A). We further used a HAMD score 17 as cut-off to stratify the BDII-D population into a mild depression subgroup and a moderate-to-severe depression subgroup. The results showed that the mSFG-PCC FC and HAMD association was nonsignificant in the mild subgroup (r = 0.20, q = 0.06) but remained significant in the moderate-to-severe subgroup (r = 0.42, q = 0.001) (Fig. 3B).

Beyond these significant findings, we further examined the associations between the identified brain FC alterations and peripheral blood markers and clinical variables in the BDII-D group. These included additional inflammatory markers (IL-6, TNF-α, and CRP), indirect inflammatory markers, psychiatric symptoms, and clinical profiles. The relationships between significant FC values and childhood adversity as well as sleep and circadian rhythm characteristics were also explored in both BDII-D individuals and HCs. However, none of these additional associations survived correction for multiple comparisons. Results are shown in the Supplementary Tables 2–12.

### Moderation analysis

Based on the partial correlational results among left caudate-insular connectivity, IL-1β, and HAMD score, we established the moderation models with IL-1β as the moderating variable, left caudate-insular connectivity as independent variables, and HAMD as the dependent variable. Table 2 showed the results of moderation analyses and model assumptions. IL-1β moderated associations between higher caudate-insular connectivity (B = 4.36, 95% CI 2.501–6.228, *p* < 0.001) and greater depressive symptoms (Fig. 2C&D).

## Discussion

We observed that higher caudate-insula connectivity, higher right tIFG-bilateral SFG connectivity, and lower mSFG-PCC connectivity were significantly associated with greater depressive symptoms. As hypothesized, IL-1β significantly moderated the association between caudate-insula connectivity and depressive severity, suggesting that inflammation may influence depression by altering striato-insular network interactions involved in affective processing. The mSFG-PCC finding initially appeared counterintuitive, however, when BDII-D individuals were stratified into mild and moderate-to-severe subgroups, the association between mSFG-PCC connectivity and HAMD scores was nonsignificant in the mild subgroup but became significant in the moderate-to-severe subgroup. This pattern suggests a potential compensatory mechanism, whereby individuals with more severe depressive symptoms may upregulate mSFG-PCC connectivity to maintain brain function, although connectivity levels remain lower than those observed HCs. In addition, lower left MFG-right lateral SOG connectivity was significantly associated with higher PANSS positive scores, highlighting a frontal-visual network mechanism in which disrupted top-down frontal modulation of visual cortical regions may contribute to the psychotic symptoms.

Increasing evidence has highlighted the role of caudate and insula in MDD and BD [36, 37]. The caudate plays a fundamental role in reward processing and the initiation of behavior, and it has been established as a core region in the pathogenesis of depression [38]. Meanwhile, the insula, particularly its left side and anterior part, serves as the brain’s primary hub for interoceptive processing and emotional regulation [39–41]. Although previous studies in BD I have frequently reported enlarged caudate volumes with higher activities and connectivity, our findings revealed lower caudate volume linked to higher FC with the left anterior insula in BDII-D. Given that BDII-D participants were characterized by a depression-dominant course with minimal hypomanic episodes, this structural profile might share a common pathophysiological substrate with MDD rather than BD I. This structural reduction, when linked to a higher FC pattern, likely drives the affective dysregulation observed in BDII-D and reflects the cumulative neurobiological impact of prolonged depressive states on striato-insular circuits. Additionally, IL-1β is a key driver of both peripheral and central immune responses [42]. Elevated levels of IL-1β have been observed in both MDD and BD and are closely linked to the behavioral and neurochemical changes that regulate human mood[43–45]. Our observation that higher IL-1β levels moderate the FC between these regions suggests a maladaptive sensitization of the striato-insular network to systemic inflammation. Specifically, under conditions of high inflammation, the caudate may become pathologically coupled with the anterior insula, collectively driving the affective dysregulation characteristic of BDII-D.

Default mode network (DMN) is a large-scale, intrinsically connected system that supports internally oriented cognition, with the mPFC and PCC serving as key hub nodes within its core architecture [46]. One seemingly counterintuitive pattern emerged in current study that BDII-D showed lower mSFG-PCC connectivity compared to HCs, yet within BDII-D, higher mSFG-PCC connectivity was associated with greater depressive severity, particularly in the moderate-to-severe subgroup. These findings are not mutually exclusive, because between-group effects reflect an overall shift in the patient distribution whereas within-group correlations capture individual differences among individuals with BDII-D. One way to interpret this pattern is that BDII-D involves a general weakening of connectivity within the DMN core, but that as depressive symptoms become more severe, the DMN core becomes more tightly coupled again within patients. In other words, the BDII-D group may be shifted toward lower mSFG-PCC connectivity overall, yet those with greater symptom burden show relatively higher connectivity than those with milder depression. This fits prior work showing that DMN findings are not always consistent in direction across studies or DMN inter-network and may reflect an internal imbalance rather than a uniform increase or decrease [47]. It also aligns with rumination-based models, which propose that more severe depression is marked by stronger engagement of internally focused, self-referential processing due to abnormal interactions between DMN circuitry and affect-related networks[48].

The observed association between lower left MFG-right lateral SOG connectivity and higher PANSS positive scores provides critical support for the eye-brain axis hypothesis [49, 50]. This finding suggested that a breakdown in the frontal-visual network serves as a core mechanism for psychotic positive symptoms in BDII-D. Interestingly, while our recent study showed higher FC in SFG-visual cortex in early-onset BD (under review), the current results showed distinct correlation direction. This patten likely reflects a compensation-to-failure model where in the earliest stages of BD, higher frontal-visual FC may represent a compensatory neuroplastic effort to regulate or ‘gate’ abnormal sensory inputs originating from a compromised retina. However, as the disorder progresses or symptoms intensify, this top-down modulation fails. The current finding, whereby lower frontal-visual connectivity is associated with higher psychotic positive symptom severity in BDII-D, is consistent with frontal-visual circuit dysfunction observed in psychosis, in which the lack of primary visual cortex involvement allows higher-order visual areas to generate hallucinations [51]. Further research is needed to clarify how retinal neurodegeneration and microvascular changes relate to structural and functional abnormalities in occipital and frontal regions in psychiatric disorders. Integrating retinal biomarkers with measures of frontal-visual circuit dysfunction may strengthen the case that impaired visual-system integrity is a primary, rather than secondary, contributor to the pathophysiology of these disorders.

Several limitations, many of which were also acknowledged in our previous work, should be noted. These include the cross-sectional design (precluding causal inference), incomplete inflammatory profiling due to the absence of blood samples in HCs, potential residual confounding from medication exposure despite adjustment for medication load, and imperfect demographic matching (particularly age). Future studies with longitudinal designs, well-matched samples, and more comprehensive phenotyping will be needed to address these issues. Additionally, although our study included sleep and circadian rhythm scales, we didn’t observe any significant correlation with altered brain FC in BDII-D. This may reflect the ROI-based nature of our analysis that the priori regions we selected may not capture the networks most directly implicated in sleep-circadian regulation, reducing sensitivity to observe such associations. Future studies using whole-brain approaches may better characterize sleep- and rhythm-related network alterations in BDII-D.

## Conclusions

Our findings suggested that depressive severity in BDII-D was driven by a complex breakdown in brain connectivity, specifically involving networks responsible for emotion and sensory processing. The inflammation, specifically higher IL-1β levels, seemed to worsen depression by disrupting the connection between the caudate and the insula, while as symptoms became more severe, the brain may try to compensate by increasing connections between mSFG and PCC.

Additionally, alterations in the frontal-visual circuit appear to be a key factor behind psychotic positive symptoms. These results highlight that BDII-D involves a unique neurobiological profile where systemic immune markers and brain-wide communication patterns interact to shape the severity of the illness.

## Supplementary Material

This is a list of supplementary files associated with this preprint. Click to download.

• Supplementarymaterials.docx

## Figures and Tables

**Table 1:** Demographic and Clinical Characteristics of Participants

Characteristic	BD II-D (N = 147)	Healthy controls (N = 150)	Cohen’d/χ2	*p*
**Sociodemographic**				
Age, mean (SD)	22.75 ± 7.31	25.58 ± 7.21	−0.389	<0.001
Sex(female/male)	109/38	144/36	1.582	0.209
Total education, mean (SD), yrs	13.75 ± 2.29	16.90 ± 3.14	−1.15	<0.001
marriage status (yes/no)	17/130	18/132	0.01	0.907
childbearing (yes/no)	14/133	9/141	1.29	0.256
**Sleep and circadian rhythm score, mean (SD)**				
ESS	11.23 ± 5.06	7.35 ± 3.96	0.86	< 0.001
PSQI	11.61 ±4.07	3.03 ± 2.33	2.59	< 0.001
MEQ	43.59 ± 8.98	42.52 ± 11.48	0.10	0.8
BRIAN	55.61 ±11.51	34.07 ± 7.42	2.23	< 0.001
Sleep	14.39 ± 3.34	8.79 ± 2.87	1.80	< 0.001
Activities	12.59 ± 3.88	7.21 ± 2.13	1.72	< 0.001
SociaLrhythm	9.42 ± 2.96	5.69 ± 1.72	1.55	< 0.001
Eating_pattern	10.31 ±3.23	6.01 ± 2.11	1.58	< 0.001
Predominant_rhythm	8.91 ±2.99	6.37 ± 2.27	0.96	< 0.001
**Childhood adversity, mean (SD)**				
CTQ total	47.26 ± 16.39	28.58 ± 5.58	1.53	<0.001
Emotional Abuse	10.6 ± 5.41	5.66 ± 1.42	1.25	<0.001
Physical Abuse	7.39 ± 3.18	5.29 ± 0.91	0.90	<0.001
Sexual Abuse	6.22 ± 2.69	5.12 ± 0.42	0.57	<0.001
Emotional Neglect	13.70 ± 6.23	7.04 ± 2.88	1.38	<0.001
Physical Neglect	9.34 ± 4.17	5.47 ± 1.32	1.26	<0.001
**Clinical characteristics, mean (SD)**				
No. of hypomanic episodes, mean (SD)	2.4 ± 2.07	-	-	-
The duration of depressive episode, mean (SD) (days)	98.54 ± 143.18	-	-	-
duration of illness, mean (SD) (yrs)	4.06 ± 3.85	-	-	-
Age at onset, mean (SD)	21.73 ± 7.04	-	-	-
**Questionnaires, mean (SD)**				
HAMD score	15.7 ± 5.48	-	-	-
HAMA score	12.95 ± 6.76	-	-	-
PANSS_P score	8.21 ± 2.08	-	-	-
PANSS_N score	7.89 ± 2.21	-	-	-
PANSS_G score	22.76 ± 3.83	-	-	-
PANSS_T score	38.86 ± 6.05	-	-	-
C-SSRS Intensity of ideation	2.42 ± 1.99		-	-
**Blood Cells, mean (SD)**				
IL-1beta	4.11 ±4.12	-	-	-
IL-6	2.67 ± 3.56	-	-	-
TNF-alpha	6.20 ± 3.56	-	-	-
CRP	2.70 ± 3.46	-	-	-
WBC	6.25 ± 1.80	-	-	-
Neutrophil to Lymphocyte ratio	1.61 ± 0.89	-	-	-
Monocyte to Lymphocyte ratio	0.24 ± 0.12	-	-	-
Platelet to Lymphocyte ratio	115.35 ± 42.14	-	-	-
**Medical characteristics**				
**Medication load index, mean (SD)**	1.5 ± 1.79	-	-	-
**Antidepressants, No.of.patients**	52	-	-	-
SNRIs	11	-	-	-
SSRIs	21	-	-	-
Agomelatine	19	-	-	-
**Mood stabilizer, No.of patients**	57	-	-	-
**Antipsychotics, No.of patients**	48	-	-	-
**Benzodiazepines, No.of patients**	26	-	-	-
**No medication, No.of patients**	75	-	-	-

**Abbrevations:** BD-bipolar type II depression; HAMD-17-item Hamilton Depression Scale; HAMA-Hamilton Anxiety Scale; PANSS-Positive and Negative Syndrome Scale; P-positive; N-negative; G-general; T-Total; C-SSRS- Columbia-Suicide Severity Rating Scale;MEQ-Morningness-Eveningness Questionnaire, ESS-Epworth Sleepiness Scale, PSQI-Pittsburgh Sleep Quality Index, BRIAN-BiologicalRhythms Interview of Assessment in Neuropsychiatry; CTQ-Childhood Trauma Questionnaire; IL-Interleukin; CRP- C-reactive protein;TNF-Tumor Necrosis Factor; WBC-white blood cell; SNRIs-serotonin and norepinephrine reuptake inhibitors; SSRIs-selective serotoninreuptake inhibitors.

**Table 2.** Ordinary Least Squares regression and moderation analysis for caudate-insula FC (X), and IL-1β (M), HAMD (Y)

	Estimate	SE	t-value	p-value	95% CI for Beta		VIF	R^2^	Adjusted R^2^	F
					lower	upper				
**Ordinary Least Squares regression**										
Intercept	19.59	2.65	7.38	0.00**	14.34	24.83		0.150	0.110	F(6,140) = 4.08, *p* = 0.00**
left caudate-insula FC	1.4	0.35	3.96	0.00**	0.703	2.103	1.071			
IL-1β	1.280	1.490	0.860	0.390	−1.66	4.22	1.069			
Education	−0.380	0.200	−1.870	0.060	−0.78	0.022	1.207			
Age	0.050	0.060	0.840	0.400	−0.073	0.182	1.241			
Sex	−0.380	0.990	−0.380	0.700	−2.338	1.581	1.04			
Medication load	0.13	0.240	0.540	0.590	−0.346	0.604	1.026			
**Moderate model**										
Intercept	20.26	2.48	8.160	0.00**	15.35	25.16		0.260	0.230	F(7,139) = 7.07, *p* = 0.00**
left caudate-insula FC	1.100	0.34	3.250	0.00**	0.430	1.764	1.11			
IL-1β	1.31	1.39	0.940	0.35	−1.441	4.062	1.06			
Education	−0.48	0.19	−2.530	0.01	−0.859	−0.105	1.22			
Age	0.06	0.06	1.070	0.29	−0.054	0.184	1.243			
Sex	−0.36	0.93	−0.390	0.70	−2.19	1.467	1.04			
Medication load	0.24	0.23	1.040	0.30	−0.211	0.681	1.037			
left caudate-insula FC:IL-1β	4.36	0.94	4.63	0.00**	2.501	6.228	1.06			

OLS: D-W = 1.76; p-value = 0.164; Moderate model: D-W = 1.81; p-value = 0.23

95% CI was estimated by bootstrap with 5000 repetitive times

* *p* < 0.05

** *p* < 0.01
